# Hemoglobin A1c, hemoglobin glycation index, and triglyceride and glucose index: Useful tools to predict low feed intake associated with glucose intolerance in lactating sows

**DOI:** 10.1371/journal.pone.0267644

**Published:** 2022-05-05

**Authors:** Rosa Elena Pérez, Cyntia Michelle González, Manuel López, Katya Vargas, Gerardo Ordaz, Ruy Ortiz

**Affiliations:** 1 Faculty of Chemical Pharmacobiology, Universidad Michoacana de San Nicolás de Hidalgo, Michoacan, Mexico; 2 Faculty of Veterinary Medicine and Zootechnics, Universidad Michoacana de San Nicolás de Hidalgo, Michoacan, Mexico; 3 Department of Medical Sciences, Division of Health Sciences, Universidad de Guanajuato, Guanajuato, Mexico; 4 National Center of Disciplinary Research in Animal Physiology and Genetics, INIFAP, Queretaro, Mexico; Sejong University, REPUBLIC OF KOREA

## Abstract

The aim of the present study was to evaluated hemoglobin A1c (HbA1c), the hemoglobin glycation index (HGI), and triglyceride and glucose (TG) index as predictive indicators for low feed intake in lactating sows due to glucose intolerance. Cactus (*Opuntia ficus-indica*) was included in sow diets as a modulating factor of glucose. Thirty-six sows were separated into three groups (Gs). Although the three groups received a conventional diet during gestation and lactation, 2.0 kg per sow per day of steam-cooked cactus (G1) and fresh cactus (G2) were added to the lactation diet as a glycemic modulating factor, with G3 serving as the control group. Glycemia was assessed via glucometer (blood glucose concentrations), HbA1c and HGI. For each indicator of glycemia the triglycerides and glucose (TG) index was evaluated. The highest blood glucose concentration was observed on day 3 of lactation (88.2 mg/dL). The average glycemic concentrations obtained from HbA1c on farrowing day (61.6 mg/dL) and day 21 of lactation (65.6 mg/dL) were lower (p<0.05) than those measured by a glucometer on the same days (71.8 and 77.7 mg/dL for farrowing day and day 21 of lactation, respectively). At farrowing, the TG index obtained from the HGI indicated that 83.0% of sows were glucose intolerant, compared to 100% according to the TG index obtained from a glucometer. At weaning, 50% of G2 did not show glucose intolerance when the TG index was calculated using the HGI, compared to 54% when it was calculated with blood glucose concentrations measured by a glucometer. All G3 sows presented glucose intolerance, regardless of the test used. The HbA1c, HGI, and TG index tests are viable alternatives to predict low feed intake due to glucose intolerance in lactating sows.

## Introduction

The close and complex relationship between nutrition-feeding (quantity and quality) and sow productivity is fundamental to the profitability of production systems [1–3]. However, independent of improvements in pig feed, the feed intake of sows during lactation has not changed substantially [4]. Regarding feed intake of lactating sows, more than 50% of hyperprolific sows have an average feed intake of less than 5.5 kg per day, which does not provide the nutrients they require [5, 6]. This low feed intake is due to glucose intolerance that sows suffer from during peripartum and lactation [7, 8]. The low feed intake of lactating sows favors greater weaning-estrus interval, less fertility and prolificacy and a higher percentage of repeated services in the following productive cycle, whose final result is the increase in non-productive days of the sow and a lower number of parities per sow per year [9–11].

To counteract the effects of glucose intolerance, it has been reported that the addition of fiber from various foods, including cactus (*Opuntia ficus-indica*), to the diet of gestating and lactating sows increases feed intake during lactation [12–15]. This is because dietary fiber favors the metabolic profile of sows [12, 15]. Specifically, the inclusion of cactus in the diet of sows stimulates insulin secretion and improved glucose reabsorption by different tissues, which are associated with lower glucose concentrations [14, 16]. In humans, it has been reported that the consumption of cooked cactus modulates glucose intolerance better than raw cactus because of the increase in the bioavailability of its nutrients, mainly soluble fiber [16]. Although the inclusion of cooked cactus in sow diets could be a more efficient means to modulate glucose intolerance, it remains unexplored. It should be noted that currently not only new nutritional strategies are required but also technologies or tools to evaluate and control metabolic changes in sows as is glucose intolerance in lactation [17, 18].

Glucose intolerance in sows has traditionally been monitored using the euglycemic-hyperinsulinemic clamp during periods of high energy demand [7, 19, 20]. However, the diagnosis of glucose intolerance using this test apart from that it is complicated in field may vary between individuals [21–24]. To solve this problem and facilitate the diagnosis of glucose intolerance, the glycated hemoglobin (HbA1c) test has been used in conjunction with the hemoglobin glycation index (HGI) and triglyceride and glucose (TG) index [25–27].

The HbA1c test is an indirect measure of average glycemia over the previous three months [28]. The advantages of HbA1c as a diagnostic test are high repeatability and pre-analytical stability; moreover, HbA1c can be measured post-prandially and has low daily variations [28, 29]. The HGI is a biomarker of population variation in HbA1c due to factors other than blood glucose concentration [30]. The HGI quantifies the magnitude and direction of inter-individual variation in HbA1c based on the difference between observed and predicted HbA1c. With respect to the TG index, increased triglyceride concentrations have been reported to correlate with muscle glucose metabolism [31]. Elevated serum and tissue triglyceride concentrations are associated with decreased insulin sensitivity [32]. Because of its relationship with glucose metabolism and insulin sensitivity, triglyceride concentration has been suggested as a useful alternative for diagnosing glucose intolerance. [26, 27, 33]. In accordance with the aforementioned precedents, this study hypothesized that the use of HbA1c, HGI, and TG index tests in swine production could be a tool to diagnose sows that will present low feed intake during lactation due to glucose intolerance. This diagnosis will help to establish nutritional strategies that maximize the feed intake of lactating sows. Therefore, the objective of this study was to evaluate HbA1c, HGI and TG index tests as predictive indicators for low feed intake in lactating sows due to glucose intolerance, taking as a modulating factor of glucose the inclusion of cactus (fresh and steam-cooked) to the sows’ diet.

## Materials and methods

### Animal care

The protocol was reviewed and approved by the Technical Scientific Committee of the Faculty of Veterinary Medicine and Zootechnics (FVMZ) of the Universidad Michoacana de San Nicolás de Hidalgo (UMSNH). Animal handling was conducted in accordance with the guidelines of the Official Mexican Standard for the Production, Care, and Use of Laboratory Animals NOM-062- ZOO-1999 [34] and those of the International Guiding Principles for Biomedical Research Involving Animals [35].

### Animals, diets, and husbandry

Thirty-six pregnant sows (TOPIGS NORVIN [TN70]; parity between 1 [n = 15], 2 [n = 12] and, 3 [n = 9] farrowings; 210±10 kg live weight on day 110 of gestation) were selected at random. The total number of animals was randomly distributed in a 3×2×2 factorial design: three groups, two tests to determine glycemia (using a glucometer and HbA1c), and two periods [gestation–prefarrowing (day 25 to 110 of gestation) and prefarrowing–weaning (farrowing day at weaning)]. During the gestation phase, the sows were housed in individual cages measuring 240×65 cm (length and width, respectively), which had a semi-automatic feeder and a pivot-type drinker. During farrowing and lactation, the sows were housed in stainless steel cages with a plastic mesh floor until weaning (21 d post farrowing). During the farrowing and lactation phases, artificial light was provided between 08:00 and 15:00, and the ambient temperature was 18–24°C. Each cage contained a heat source for piglets. Farrowing occurred naturally on day 114±0.53 of gestation. The sows had a litter size of 12.1±1.2 piglets, comprising: 11.1±0.4 live births, 1.1±0.6 stillbirths, and 0.2±0.03 mummies. Litters were balanced by 10 piglets within the first 48 h post farrowing. Piglets that died during lactation were not replaced. During lactation, 31 casualties were recorded (8.5% pre-weaning mortality): 25 casualties were caused by crushing and six by diarrhea during the first and third weeks of lactation, respectively.

For the purposes of this study, the 36 sows were randomized into three groups (Gs) of 12 individuals according to parity and live weight at day 110 of gestation: parity 1 n = 5, parity 2 n = 4 and, parity 3 n = 3; per G. In G1, sows were fed a conventional diet (CD) during gestation, supplemented with steam-cooked cactus during lactation (2.0 kg per sow per day). In G2, sows were fed a CD during gestation, supplemented with fresh cactus during lactation (2.0 kg per sow per day). In G3, the control group, sows were fed only a CD. The three experimental diets were analyzed in duplicate for dry matter (Method 944.01, AOAC) [36], crude protein (Method 976.05, AOAC) [36], ether extract (Method 30–25, AACC) [37], and ash (Method 08–01, AACC) [37], while fiber was analyzed as described by van Soest et al. [38]. Tables 1 and 2 show the ingredients and nutritional compositions of the diets used.

**Table 1:** Ingredients and nutritional composition of diets.

Ingredients (%)	Diets		
	Gestation	Lactation	
Sorghum	82.40	64.95	
Soybean meal	6.00	10.00	
Canola meal	6.12	18.52	
Mono and dicalcium phosphate	1.18	0.53	
Calcium carbonate	1.40	1.24	
Soy oil	2.20	3.85	
Lysine-HCl	0.05 / 0.04	0.09 / 0.08	
DL-methionine	0.09	0.15	
Salt	0.30	0.30	
Vitamins and minerals premix^a^	0.20	0.25	
Calculated nutrient levels (%)		without OFI	whit OFI
Metabolizable energy (MJ/kg)	13.8	13.8	13.8
Crude protein	12.5	17.5	17.4
Crude fat	3.7	4.5	4.4
Fiber	3.1	4.3	4.5
Moisture	12.0	12.0	12.8
Ash	10.0	10.0	9.9
Calcium	1.4	1.2	1.35
Phosphorus	0.64	0.67	0.66
Lysine	0.52	0.95	0.94
Methionine + cysteine	0.43	0.59	0.59

^a^ Contribution per kg of feed: Cu 30 mg; Fe 160 mg; Zn 160 mg; Mn 55 mg; Se 0.5; Cr 0.2 mg; Vitamin A 14.200 IU; Vitamin D_3_ 2800 IU; Vitamin E 125 mg; Vitamin K_3_ 5 mg; Vitamin B_1_ 2.4 mg; Vitamin B_2_ 8.7 mg; Vitamin B_6_ 4.5 mg; Vitamin B_12_ 0.05 mg; Pantothenic acid 35 mg; Acid folic 6 mg.

**Table 2:** Analyzed values for cactus (*O*. *ficus-indica*) and major nutrients used in diets.

Nutrient levels (%)	Cactus		Sorghum	Soybean meal	Canola meal
	Precooked	Fresh basis			
Metabolizable energy (MJ/kg)	9.2	9.1	14.6	14.8	14.7
Crude protein	6.5	5.6	8.6	44.2	34.8
Crude fat	0.2	0.2	2.7	5.6	8.72
Fiber	26.5	28.8	2.1	5.7	9.1
Moisture	87.1	88.6	13.4	92.0	92.5
Ash	24.0	24.5	1.2	5.8	6.5
Calcium	--	--	0.02	0.27	0.62
Phosphorus	--	--	0.03	0.64	1.09
Lysine	--	--	0.20	2.81	1.43
Methionine + cysteine	--	--	0.16	0.62	0.67

During the gestation phase, all three groups were fed a single ration at 07:30 (2.5 and 3.0 kg per sow per day during the first two-thirds and last third of gestation, respectively). Steam-cooked (G1) and fresh (G2) cactus were added to rations at 07:30 from the farrowing day until weaning. The commercial feed at lactation was supplied *ad libitum*. To stimulate a high commercial feed intake, the feed was supplied three times a day, at 08:00, 12:00, and 16:00. The rejected feed was weighed every following morning before supplying the cactus, except on blood sampling days, in which the feed was withdrawn at 20:00 to facilitate a 10-h fasting period.

The cactus cladodes had an average age of 90 d and were collected from the Posta Zootécnica plot of the FVMZ-UMSNH. As the cladodes of *O*. *ficus-indica* lack spines, they were cut into 1.0-cm^3^ pieces, either to be fed fresh (G2) or steam-cooked (G1). The cactus pieces were steamed in a container for 4 min at 100°C, then placed in a container with water at a temperature of 7°C, stored, and refrigerated at 3°C until they were added to the G1 diet.

Productivity indicators of sows with diets supplemented with cactus were not incorporated into this study, as they have already been discussed in other studies [14, 39]. Instead, this research focused on evaluating blood glucose, HbA1c, and triglyceride concentrations and associating them with HGI and TG index to diagnose glucose intolerance in sows and determine its relationship with low feed intake during lactation. Regarding the animals used for experimentation, once the work was completed, these animals were incorporated into the pig production system of the FVMZ-UMSNH for future research.

### Blood sampling

For the determination of blood glucose concentration in the sows, blood samples were collected at 06:00 h (10 h fasting) as follows. Preprandial blood glucose samples were taken per sow per group on days 25, 45, 65, 85, and 110 of gestation, farrowing day (day 114±0.53 of gestation), days 0, 3, 7, 14, and 21 of lactation, and day 7 post weaning. On these days, the auricular vein was punctured in each sow with a sterile hypodermic needle (18G×1″) to collect a drop of blood (approximately 0.6 μL per sample). The blood drop was then deposited on a test strip to read the corresponding whole blood glucose concentration in a glucometer for human use. The choice to take blood samples from the atrial vein was based on its accessibility, which did not require the animals to be physically restrained and minimized their stress. Additionally, it has been reported that the venous blood glucose concentration is a good indicator for clinically testing blood glucose because of its high stability and few interference factors [40].

For the determination of HbA1c and triglyceride concentration in the sows, preprandial blood samples were taken per sow per group on farrowing day (day 114±0.53 of gestation) and day 21 of lactation (weaning day) at 06:00. On each of these days, a trap cable was fastened to the upper jaw of each sow to collect the respective 5-mL blood samples, which were extracted from the jugular vein using a syringe with an 18G×2″ hypodermic needle. After collection, each sample was divided into two aliquots in BD Vacutainer^™^ tubes (2.5 mL per tube): one tube without an anti-coagulant to measure HbA1c in whole blood and another with an anti-coagulant (EDTA K2) to measure triglycerides in plasma. Samples were stored at 4°C until the first aliquots were sent to the laboratory to measure HbA1c. The second aliquots were centrifuged (10,000 × g for 10 min at 4°C) to measure triglycerides. The plasma to measure triglycerides was stored and frozen at −20°C until analysis.

### Metabolite analysis

To measure blood glucose concentration, a glucometer for human use (Accu-Check Performa^®^, Roche Diabetes Care GmbH, Mannheim, Germany) was used according to the methodology established by Pérez et al. [41]. Triglyceride measurements performed using adapted enzymatic methods on a Cobas c111^®^ (Roche, Basel, Switzerland). The reagent used was TRIGL (ref. 04 657 594 190, USA), with a sensitivity of 9.0 mg/dL; the intra- and inter-assay coefficient of variation was <8.0 and <14.0% at 600 mg/dL, respectively.

To measure HbA1c levels, high-resolution liquid chromatography was used [42]. This is an international technique endorsed by the Diabetes Control and Complications Trial (DCCT), using an automated analyzer certified by the National Glycohemoglobin Standardization Program (NGSP). The HbA1c levels measured in the laboratory are expressed as percentages. The conversion from HbA1c percentage to glycemic concentration (mg/dL) was performed using the following equation established by Nathan et al. [43]:

Glycemicconcentration(mg/dL)=28.7*HbA1c(%)−46.7. (1)

### Statistical analysis

All statistical analyzes were performed with SAS version 9.4 (SAS Inst. Inc., Cary, NC, USA). Prior to data analysis, the normality of the distribution and homogeneity of the variance of the data were determined using PROC UNIVARIATE. The Shapiro-Wilk test was used to determine normality, while the Bartlett test was used to determine homogeneity. In the case of non-normality, parameters were normalized by Ln transformation prior to analysis to generate a normal distribution. Data were analyzed using ANOVA according to the established design (factorial 3×2×2) through repeated measurements (PROC MIXED) [44], with sow nested within the group as a source of random variation and group, reproductive phase, test, sampling day, and the interaction group × reproductive phase and group × reproductive phase × test as sources of fixed variation. Litter size is incorporated into the model as a covariate in order to reduce the existing variation with respect to the number of fetuses on the behavior of biochemical indicators. The least squares mean method was used to determine the difference between the means with α≤0.05.

Sow blood glucose and HbA1c data were used to estimate the linear relationship between the two parameters, as described in previous studies [45–47]. The predicted HbA1c level for each sow was calculated by inserting the blood glucose concentration into the following linear regression equation:

HbA1c=3.8070+0.0196*bloodglucoseconcentration(mg/dL);

*r* = 0.608; p<0001. The HGI was calculated by subtracting the measured HbA1c from the predicted HbA1c concentrations as follows:

HGI=measureHbA1c(mg/dL)−predictedHbA1c(mg/dL)

[45].

To obtain the TG index, we used the following calculation established by Guerrero-Romero et al. [27]:

TGindex=[Ln(fastingtriglycerides)(mg/dL)×fastingglycemia(mg/dL)/2], (2)

where Ln is the natural logarithm.

Due to the variation that prevails in the concentration of blood glucose measured by glucometer and the glycemia measured by HbA1c and HGI. The TG index was calculated using the blood glucose concentration or glycemia obtained by these methods. This made it possible to compare variations in the TG index with respect to the method of obtaining glycemia and establish the best means of predicting glucose intolerance in sows. To determine the effect of the TG index on sow feed intake during lactation, linear regression coefficients (β0 and β1) were estimated (PROC REG), not considering the evaluation group.

Sows were categorized into tertiles according to their HGI and TG indexes: low (≤ 0.263), moderate (0.264 to 0.091), and high (≥0.092) HGI; and low (≤8.3), moderate (8.4 to 8.7), and high (≥8.8) TG index. The use of a tertile classification system was consistent with previous studies [45, 47]. The values in the tables and figures are presented as the minimum squares ± standard error of mean (SEM).

## Results

### Blood glucose and HbA1c concentrations during gestation and lactation phases with no cactus intake

**Fig 1:**
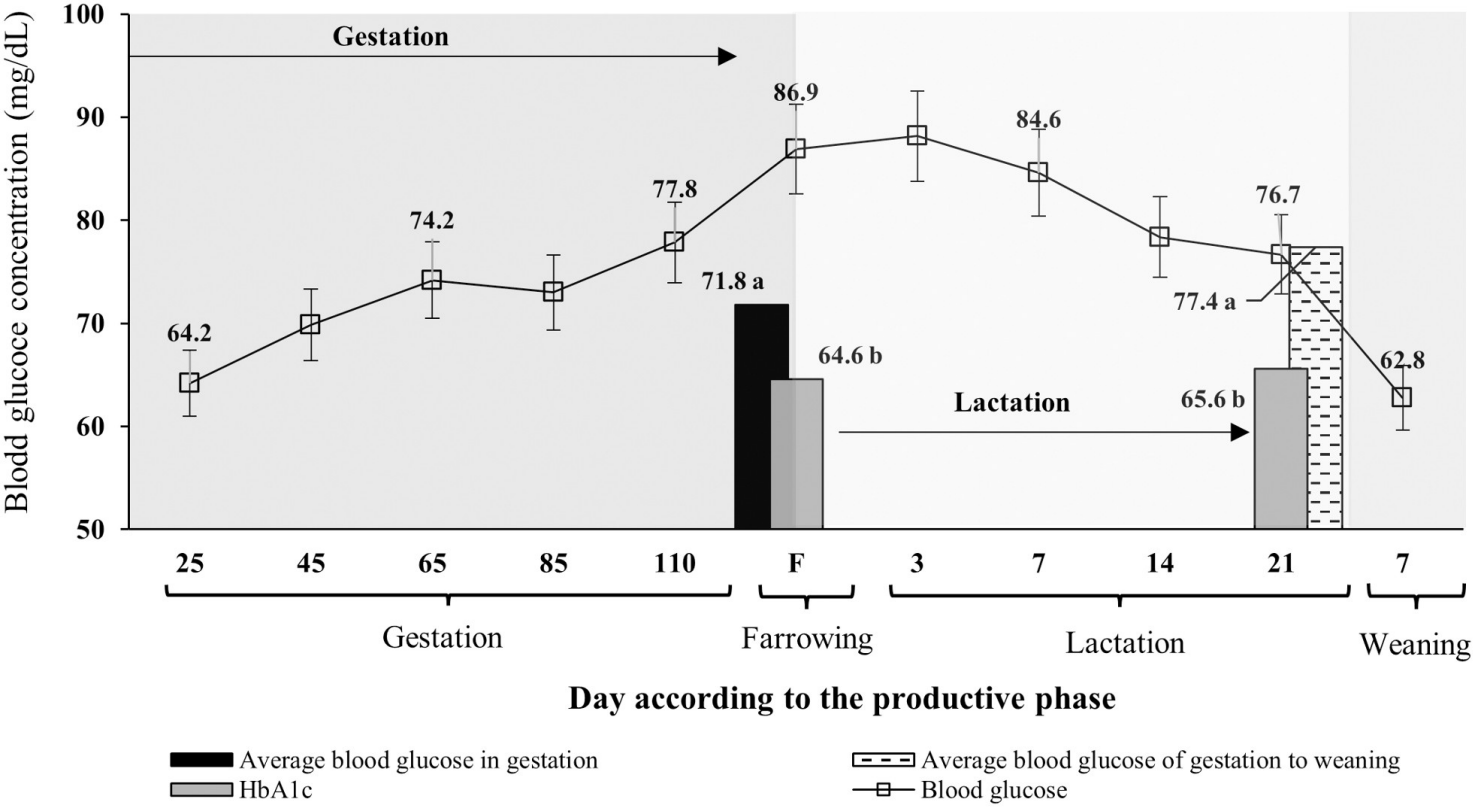
Dynamics of preprandial blood glucose of sows during the gestation to weaning phases and HbA1c on the farrowing and weaning day, regardless of cactus intake during lactation. Each point or bar represents the mean ± standard error of mean (SEM; n = 36 sows).

Blood glucose concentrations (obtained using the glucometer) made it possible to determine the dynamics of sows’ glucose from day 25 of gestation to weaning, as well as during the phases of weaning, gestation, farrowing, and lactation. This was done for all three groups of sows (Fig 1). It was observed that as gestation progressed, blood glucose concentrations per day increased (p<0.05); this increase was more accentuated on farrowing days than on other days (Fig 1).

On the farrowing day and day 3 of lactation, blood glucose concentrations were at their highest (p<0.05; Fig 1). After day 3 of lactation, blood glucose concentrations decreased until reaching 76.7 mg/dL on the day of piglet weaning (day 21 of lactation); on day 7 post weaning, blood glucose concentrations were at their lowest (Fig 1). There was no significant difference in average HbA1c values between the farrowing day and weaning day (p>0.05; Fig 1). HbA1c values were lower (p<0.05) than the average preprandial blood glucose concentrations obtained using the glucometer in the gestation–prefarrowing and prefarrowing–weaning phases (Fig 1).

### Blood glucose and HbA1c concentrations during gestation and lactation phases with cactus intake

**Fig 2:**
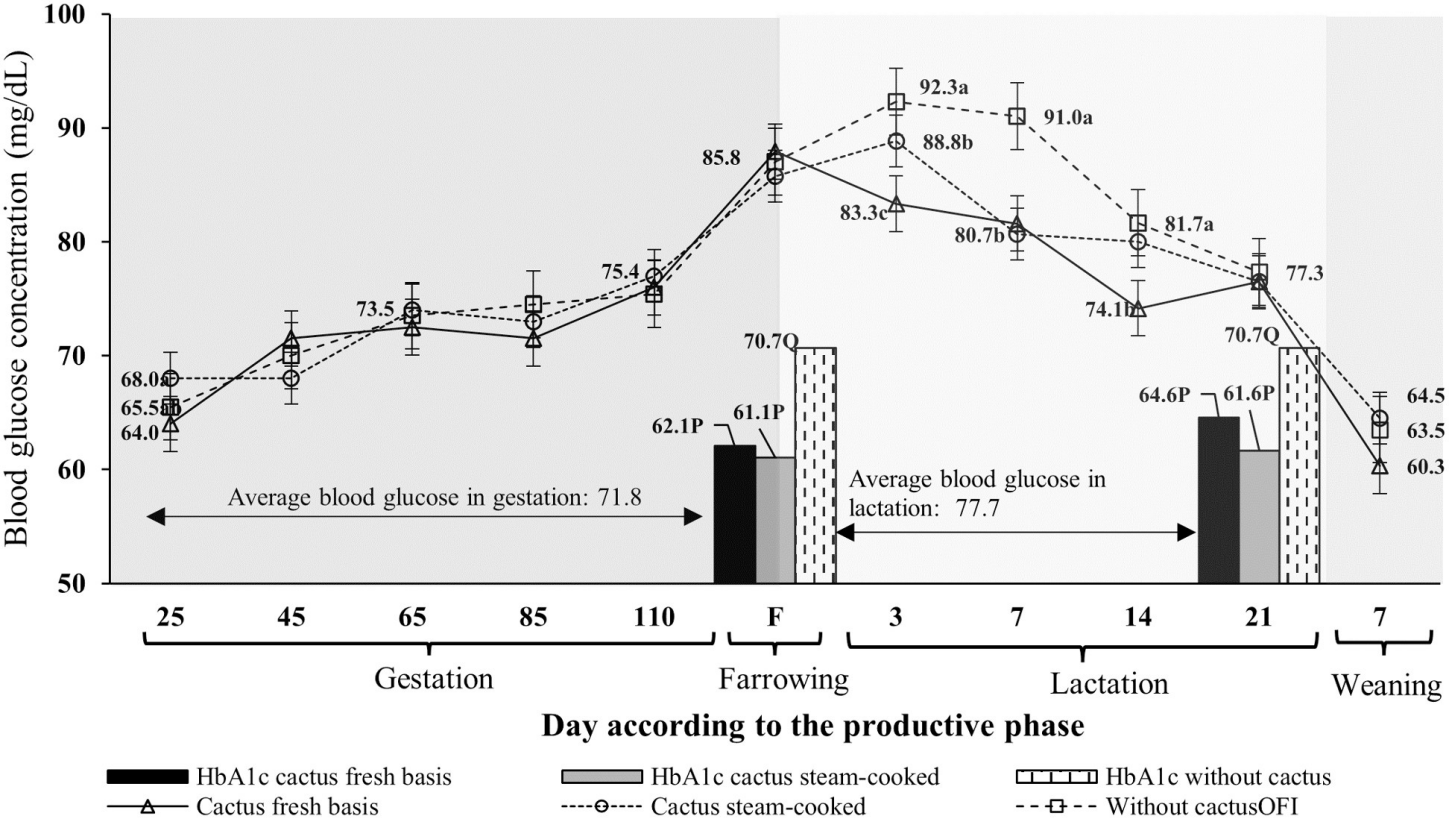
Dynamics of preprandial blood glucose of sows during the gestation to weaning phases and HbA1c on the farrowing day and weaning day, with cactus intake during lactation. Each point or bar represents the mean ± standard error of mean (SEM; n = 12 sows.

Regarding the dynamics of preprandial blood glucose concentrations in the three groups of sows analyzed, no differences were found (p>0.05) during gestation: blood glucose concentrations increased (p<0.05) in the three groups as gestation progressed (Fig 2). Differences (p<0.05) in blood glucose concentrations were observed between groups in the pre-farrowing–weaning phase (Fig 2); the G1 and G2 sows (fed steam-cooked and fresh cactus, respectively) showed a lower blood glucose concentration (p<0.05) than the G3 sows (control). The highest blood glucose concentration (p<0.05) was found in G1 and G3 on day 3 of lactation (Fig 2). After day 3 of lactation, blood glucose concentrations decreased (p<0.05) to a greater extent in G1 and G2 than in G3 (Fig 2), with this decrease in blood glucose being more pronounced in G2 on day 14 of lactation. At the end of lactation (21 days post-farrowing), blood glucose concentrations in the three groups were equal (p>0.05; Fig 2).

The average HbA1c concentrations were lower (p<0.05) than the average blood glucose concentrations in equivalent groups and reproductive phases (Table 3). For example, the average blood glucose concentration measured by the glucometer during the gestation–pre-farrowing phase in G3 was 71.8 ± 2.9 mg/dL. In the same phase and group, the HbA1c test measured a lower (p<0.05) average glycemic concentration of 61.4±3.7 mg/dL (Table 3). The cactus intake did not affect the glycemic concentration recorded by the HbA1c test in G1 and G2. The glycemia (HbA1c) in the groups of sows that consumed cactus showed similar values (p>0.05) within each reproductive phase (Table 3). In G3, the HbA1c test recorded the highest glycemic concentration of 70.7 mg/dL in the pre-farrowing–weaning phases (Table 3).

**Table 3:** Least squares mean (± SEM) for the average preprandial blood glucose concentration (mg/dL) and HbA1c (mg/dL) according to the group and production phase of the sows.

Group (G)	Test	Production phase	
		Gestation-Prefarrowing	Prefarrowing-Weaning
G1, cactus precooked	Glucometer	72.1^aP^ ± 2.9	83.3^aP^ ± 1.4
	HbA1c	61.0^b1^ ± 3.7	61.4^b1^ ± 4.0
G2, cactus on fresh basis	Glucometer	70.7^aP^ ± 2.9	81.3^aP^ ± 1.4
	HbA1c	61.9^b1^ ± 3.7	64.3^b1^ ± 4.0
G3, control	Glucometer	71.8^aP^ ± 2.9	85.2^aQ^ ± 1.4
	HbA1c	61.4^b1^ ± 3.7	73.2^b2^ ± 4.0

Literals ^a, b^ indicate differences (p<0.05) between glucometer and HbA1c within group and phase.

Literals ^P, Q^ indicate differences (p<0.05) in glycemic between groups within phase.

Numerals ^1, 2^ indicate differences (p<0.05) of HbA1c between groups within phase.

### HGI and TG index in the farrowing and lactation phases

The TG index was calculated using blood glucose concentrations measured by the glucometer, glycemia of the HbA1c and, HGI. On both the farrowing and weaning day, a higher (p<0.05) TG index (Ln) was obtained using glycemia calculated from the HGI (p<0.05) than using blood glucose concentrations measured by the glucometer or glycemia from the HbA1c test (Table 4).

**Table 4:** Least squares mean (± SEM) for the average values of the triglyceride and glucose index (Ln) according to: a, the method used to obtain glycemia (mg/dL) concentrations; b, according to the group and; c, in relation to HGI tertiles.

	a: Triglyceride and glucose index according to the method used to obtain glycemia					
	*Farrowing*			*Weaned*		
Glucometer	8.7 ± 0.02^a1^			8.5 ± 0.02^b1^		
HbA1c	8.3 ± 0.02^a2^			8.0 ± 0.02^b2^		
HGI	8.9 ± 0.02^a3^			8.6 ± 0.02^b1^		
	b: Triglyceride and glucose index according to the group^&^					
	*Farrowing*			*Weaned*		
G1, steam-cooked cactus	8.9 ± 0.04^a1^			7.8 ± 0.04^b1^		
G2, fresh basis cactus	8.8 ± 0.04^a1^			7.9 ± 0.04^b1^		
G3, Control	8.9 ± 0.04^a1^			8.4 ± 0.04^b2^		
	c: Triglyceride and glucose index in relation to HGI tertiles^&^					
	*Farrowing*			*Weaned*		
	Low HGI	Moderate HGI	High HGI	Low HGI	Moderate HGI	High HGI
G1, steam-cooked cactus	--	8.6 ± 0.06^a1^	9.0 ± 0.06^b1^	7.6 ± 0.10^c1^	7.7 ± 0.05^c1^	8.0 ± 0.10^d1^
G2, fresh basis cactus	--	8.7 ± 0.06^a1^	8.9 ± 0.06^a1^	7.6 ± 0.10^b1^	7.9 ± 0.05^c1^	8.1 ± 0.10^d1^
G3, Control	8.8 ± 0.10^a^	8.9 ± 0.07^a2^	9.0 ± 0.06^a1^	7.9 ± 0.10^b2^	8.5 ± 0.07^c2^	8.7 ± 0.06^a2^

^&^ The glycemic concentrations (mg/dL) used to determine the index were estimated using the HGI.

Literals ^a, b, c, d^ indicate differences (p<0.05) within the row.

Numerals ^1, 2, 3^ indicate differences (p<0.05) within the column.

Estimated with glycemia obtained using the HGI, regardless of the tertile, all groups presented a TG index ≥8.8 on the day of farrowing and a TG index ≤8.4 on the day of weaning (Table 4). During lactation, G3 sows presented the highest TG index (p<0.05). When evaluating the TG index (glycemia from the HGI) per group in relation to HGI tertile, the same pattern was found. On the farrowing day, the three groups presented the highest TG index in the middle and high HGI tertile (Table 4). On the day of weaning, G1 and G2 presented lower TG index values in the middle and high HGI tertile than G3 (Table 4).

At farrowing, HbA1c, TG index, preprandial blood glucose, and triglyceride concentrations were similar (p>0.05) across all three groups (Table 5). However, HbA1c, TG index, and preprandial blood glucose values were higher (p<0.05) on the weaning day. Triglyceride concentrations were lower on the farrowing day compared to the weaning day in the three groups analyzed (Table 5).

**Table 5:** Least squares mean (± SEM) for the mean values of HbA1c (%), triglyceride and glucose index (TG, Ln), preprandial blood glucose (mg/dL) and triglycerides (mg/dL) in the farrowing day and weaning day according to the group and phase.

Group (G)	Phase	HbA1c	TG index^&^	Blood glucose*	Triglycerides
G1, cactus precooked	Farrowing	5.2 ± 0.12^a^	8.8 ± 0.08^a^	71.5 ± 6.4^a^	45.9 ± 3.1^a^
	Weaned	5.4 ± 0.12^b^	8.5 ± 0.08^b^	79.8 ± 6.4^b^	34.4 ± 3.1^b^
G2, cactus on fresh basis	Farrowing	5.2 ± 0.12^a^	8.8 ± 0.08^a^	71.0 ± 6.4^a^	48.8 ± 3.1^a^
	Weaned	5.4 ± 0.12^b^	8.5 ± 0.08^b^	81.2 ± 6.4^b^	36.1 ± 3.1^b^
G3, control	Farrowing	5.3 ± 0.12^a^	8.9 ± 0.08^a^	74.2 ± 6.4^a^	49.8 ± 3.1^a^
	Weaned	5.8 ± 0.12^c^	8.9 ± 0.08^a^	100.7 ± 6.4^c^	45.3 ± 3.1^a^

^&^ Determined with the glycemia of the HGI;

*Determined by glucometer.

Literals ^a, b, c^ indicate differences (p<0.05) within the column for each indicator.

**Fig 3:**
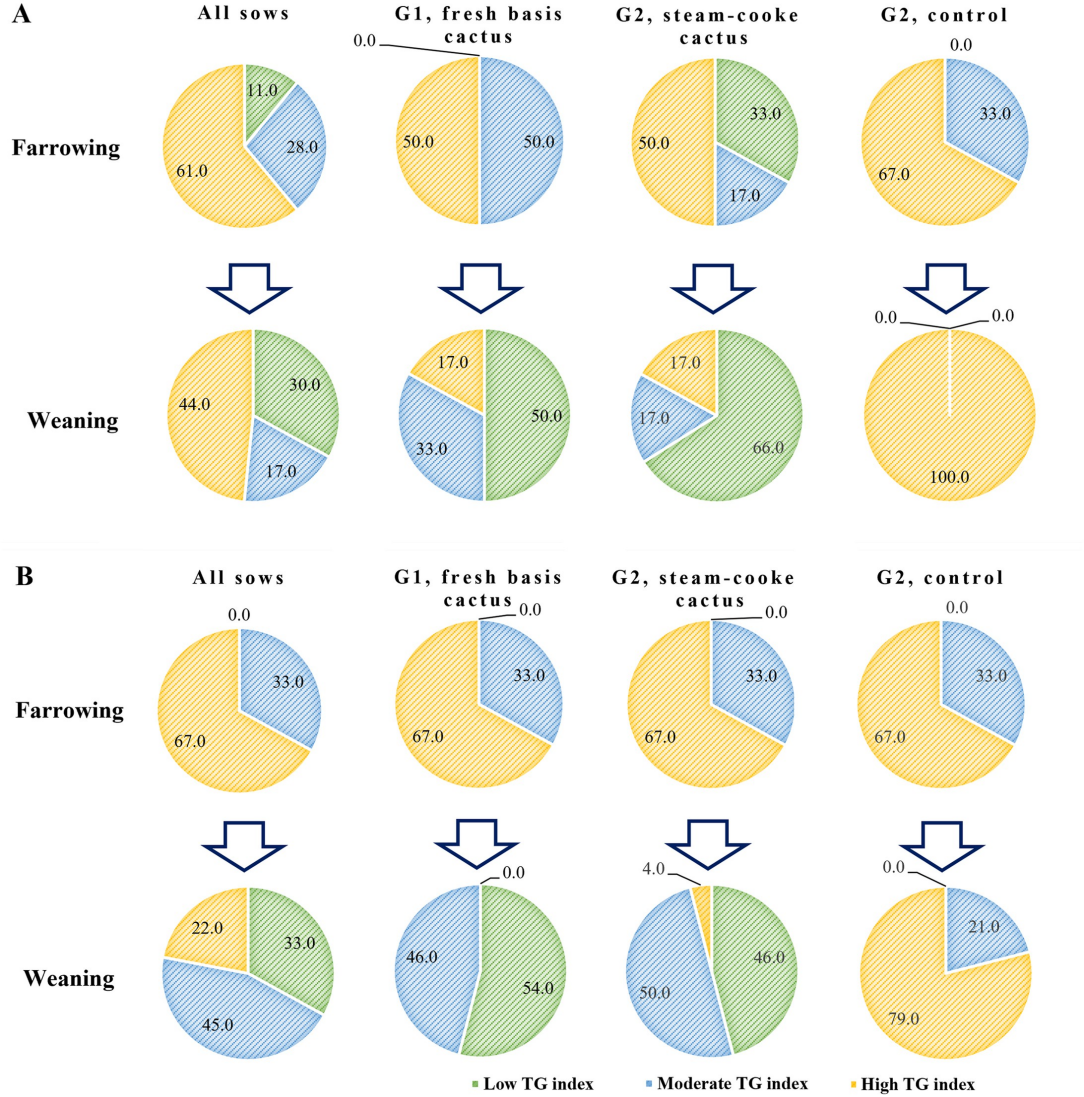
Proportion of sows with glucose intolerance according to the classification of the triglyceride and glucose index calculated using glycemia from the HGI (A) and blood glucose concentrations measured by the glucometer (B) per group and reproductive phase.

Of the sows with a low TG index, 17.0% were detected on the farrowing day. The remaining sows showed a moderate or high TG index, regardless of the group evaluated (Fig 3a). However, no low TG index derived from blood glucose concentrations measured by the glucometer was detected (Fig 3b). On the day of farrowing, 67.0% of the sows showed a high TG index, and 33.0% presented a moderate TG index, regardless of the group analyzed (Fig 3b).

On the day of weaning, the proportion of sows that presented a moderate or high TG index (glycemia derived from the HGI) was 72.0%, regardless of the group (Fig 3a). On the same day, 67.0% of sows had a moderate or high TG index (blood glucose concentration measured by the glucometer) (Fig 3b). Both G1 and G2 showed changes in the TG index (glycemia derived from the HGI) from farrowing to weaning. This was especially pronounced in G1, where 50% of exhibited a low TG index at weaning. The opposite occurred in G3: 83.0% and 17.0% of the weaned sows presented high and moderate TG index (glycemia derived from the HGI), respectively, while 79.0% and 21.0% exhibited high and moderate TG indices (blood glucose concentrations measured by the glucometer), respectively (Fig 3a and 3b).

When the average total feed intake per sow was analyzed according to the TG index and group during the 21 days of lactation, sows that showed low TG index were those that presented the highest feed intakes (p<0.05; Table 6); this result was obtained with the TG index calculated using blood glucose concentration measured by the glucometer. However, when the TG index was calculated using glycemic concentration derived from the HGI, feed intake was ≥4.9 kg per sow per day with a low TG index (Table 6). Regarding feed intake of sows in relation to moderate and high TG index, the same behavior was observed: TG index calculated using glycemia measured by the glucometer correlated with higher (p<0.05) feed intake than those using glycemia derived from the HGI (Table 6).

**Table 6:** Least squares mean (± SEM) for the average values of total feed intake* (kg per sow per day) during the lactation phase according to the triglyceride and glucose index obtained by the glycemia of the HGI or blood glucose concentration reported by the glucometer.

Triglyceride and glucose index	Tertil	General mean	Grupo (G)		
			G1, steam-cooked cactus	G2, fresh basis cactus	G3, Control
Glycemic of the HGI	Q1, Low	5.6^a^ ± 0.16	5.9^a2^ ± 0.20	5.2^a1^ ± 0.26	--
	Q2, Moderate	3.9^ab^ ± 0.23	3.9^b1^ ± 0.46	3.6^b1^ ± 0.46	4.2^a1^ ± 0.23
	Q3, High	2.8^b^ ± 0.23	--	2.7^c1^ ± 0.32	2.9^b1^ ± 0.32
Blood glucose by glucometer	Q1, Low	5.5^a^ ± 0.35	6.2^a2^ ± 0.52	4.9^a1^ ± 0.48	--
	Q2, Moderate	4.6^b^ ± 0.35	5.7^a2^ ± 0.50	4.2^a,b1^ ± 0.52	3.8^a1^ ± 0.77
	Q3, High	3.6^b^ ± 0.89	--	3.8^b1^ ± 0.50	3.4^a1^ ± 0.39

* = Commercial feed plus cactus consumption: fresh base cactus (0.529 ± 0.09 kg per sow per day), steam-cooked cactus (1.305 ± 0.09 kg per sow per day).

Literals ^a, b^ indicate differences (p<0.05) within the column for each interval.

Numerals ^1, 2^ indicate differences (p<0.05) between groups within the row.

**Fig 4:**
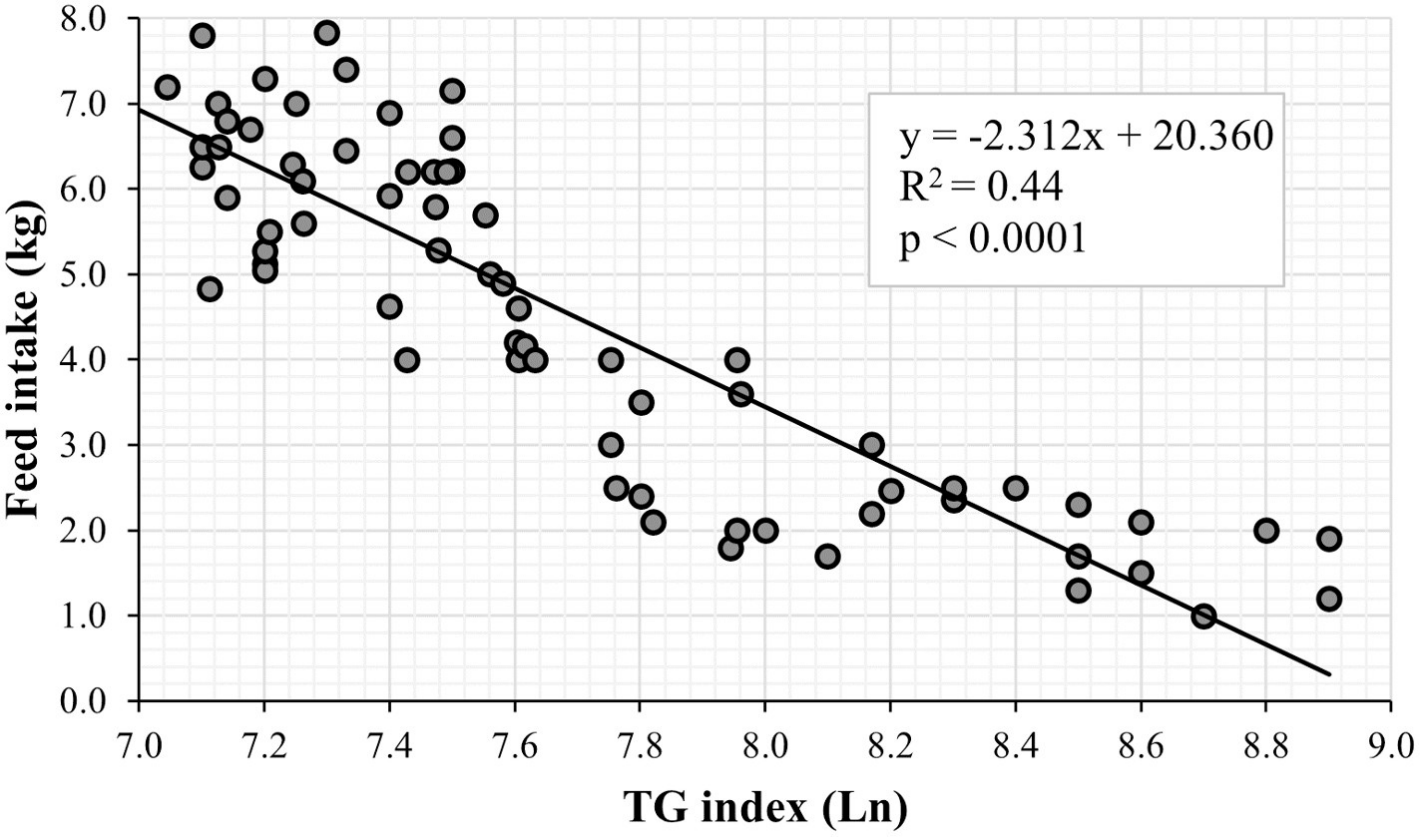
Regression line for feed intake in relation to the triglyceride and glucose (TG) index.

Finally, the voluntary feed intake of sows (regardless of group), estimated through linear regression, showed that for each point (Ln) of increase in the TG index (derived from the HGI), lactating sows reduced their feed intake by 2.31 kg (β1 = -2.312; p<0.0001; Fig 4).

## Discussion

HbA1c quantify glycemic concentration of the long period, as glucose remains bound to hemoglobin during the life of red blood cells. Therefore, HbA1c concentrations reflect the average glycemic concentration over the last three months. In pregnant women, it has been reported that HbA1c concentrations increase two- to threefold in response to the rapid growth of the fetus and placenta [46]. In most mammals, this is associated with high energy demand in the last third of gestation for the development of the fetus and milk production. This demand favors an increase in the concentration of glucose and free fatty acids, which is reflected in higher HbA1c concentrations [21]. These implications are important for monitoring glucose levels in individuals with glucose intolerance (including gestational diabetes). The quantity of glycation (formation of a stable ketoamine) could be helpful for the diagnosis of glucose intolerance in sows during late gestation and lactation [48].

It has been established that sows experience glucose intolerance from day 85 of gestation and during lactation, which limits their voluntary feed intake and affects economic indicators of swine production systems [2, 17]. The highest concentration of blood glucose was found on farrowing day and during the first week of lactation (Fig 1), which has already been reported in other studies [17, 49]. However, the HbA1c concentration at farrowing and weaning was lower than the average blood glucose concentration measured by the glucometer during gestation and lactation, a pattern also observed on the day of sampling for the determination of HbA1c and in G1 and G2 (Fig 2). It has been reported that the diagnosis of glucose intolerance based on these tests (glucometer or HbA1c) can differ significantly [23, 28, 50]. One of the main causes of the differences in the diagnostic specificity of each test is the inter-individual variation in the quantitative relationship between HbA1c and preprandial blood glucose concentration on the day of sample collection [24].

Lower glycemic concentrations have been reported [21] on day 2 post farrowing in sows of the Landrace breed than in leaner genotypes, such as Duroc, Yorkshire, and synthetic genetic lines. Similarly, the other factors influencing inter-individual variations in glycemic concentration in sows include physical condition, age, genotype, litter size, suckling intensity, and feeding quantity/quality [2, 17]. According to the findings of this study, using sows of the same genotype, factors such as age could be the primary source of this inter-individual variation. Since, e.g., the deposition of fat-free and fatty tissue in sows of the first parity differs from that in sows of the second and third parity. This has a direct effect on the reabsorption of glucose by different tissues [51].

It has been suggested that interindividual differences in the lifespan of red blood cells or other biological factors may influence HbA1c concentration, independent of the effect of blood glucose concentration [52]. Therefore, the concomitant use of the quantitative variation between HbA1c and preprandial blood glucose has been proposed to predict complications and diagnose glucose intolerance [53]. In addition to being influenced by glycemic concentrations, HbA1c can also be associated with individual differences in biological factors that influence the glycation of non-enzymatic proteins (such as age, body weight, or genetics) and the life cycle of red blood cells [54]. Given this scenario, the HGI has been developed as a method that quantifies the inter-individual variation in glycation that results in discrepancies between preprandial glycemia and HbA1c [47].

In this study, the inter-individual variation in glycation was corroborated by evaluating glucose intolerance using the TG index (Fig 4). This index demonstrated variation when using glycemic concentration obtained from the glucometer, HbA1c, and HGI. The TG index (regardless of the group) calculated using glycemia from the HGI presented higher values, both on the farrowing day and at weaning, than the TG index determined using blood glucose concentrations derived from the glucometer or HbA1c (Fig 4).

It has been determined that, for the diagnosis of glucose intolerance, the TG index must be ≥8.8 [26, 27]; the index classified as high in this study was in accordance with this reference value (≥8.8). However, owing to the feed intake of the lactating sows, the moderate TG index (8.4–8.7) was also considered as a diagnostic criterion for glucose intolerance (Tables 5 and 6), regardless of glycemia derived from the HGI or the preprandial blood glucose concentration. A high correlation (r = 0.61; p<0.001) has been reported between triglyceride concentration and decreased insulin sensitivity [32].

HGI is considered a biomarker of population variation in HbA1c due to factors other than the preprandial glycemic concentration [55]; therefore, people with a high HGI have a high risk of glucose intolerance [56]. In this context, a high degree of non-enzymatic glycation of intra-cellular proteins may increase the risk of glucose intolerance. In the case of lactating sows, a high HGI resulted in a high TG index. A moderate or high TG index increases the risk of low feed intake during lactation (Table 6 and Fig 4), a phenomenon that is related to the effect of glucose intolerance [7]. In this study, the TG index (divided into tertiles) and its relationship with sow metabolism (blood glucose, HbA1c, and triglycerides) and feed intake during lactation (Table 6) showed that more than 80.0% of the sows were classified as having a moderate or high TG index on the farrowing day. This result provides a guideline to establish that 80.0% of sows would present low feed intake during lactation, which was confirmed in this study (Table 6 and Figs 3 and 4).

According to what has been described above, it can be established that the TG index calculated using HGI can be a viable tool for the diagnosis of “problem” sows during lactation (low feed intake). This would help to implement nutritional strategies to counteract the effects of glucose intolerance on feed intake of lactating sows—aspects that were observed when adding cactus to the diet of these animals during the lactation phase. The sows that consumed cactus during lactation (G1 and G2) presented lower glycemia and triglyceride concentrations on the day of weaning (Table 5) than the control (G3). Similarly, at weaning, the groups that consumed cactus presented the highest percentage of sows (≥34.0%) with a low TG index (≤8.3). This implies that this percentage of sows does not present a risk of low feed intake during lactation associated with glucose intolerance (Table 6). This behavior was not observed in G3; in this group, 100% of the sows showed a moderate or high TG index at the end of gestation (Fig 3a).

The effect of cactus has already been studied in lactating sows [14, 39]. The regulation of glycemia due to cactus intake prevents lactational hypophagia caused by glucose intolerance. However, its effect on the metabolism of sows has not been verified using the TG index estimated from HbA1c and the HGI as a diagnostic tool. According to the results of this study, it could be established that, with the use of cactus, between 34.0% (G1) and 50.0% (G2) of the sows did not present moderate or high TG indices; that is, they were not glucose intolerant, and, therefore, feed intake was not affected during lactation (Figs 3 and 4). This is corroborated by the average feed intake per sow per day: the sows that were not glucose intolerant consumed more than 5.0 kg of feed per day (Table 6 and Fig 4). However, in accordance with this discussion, calculating the TG index from the estimated glycemia of the HGI has greater precision than when the index is estimated with glycemia measured by the glucometer (Fig 3).

This study has several strengths: the use of a glucometer and the TG index as indirect indicators of glucose intolerance, a homogeneous group of animals, the exclusion of additional factors that might affect the replacement of red blood cells, and the use of a rigorously standardized HbA1c assay. However, this study also has some limitations: firstly, daily variations in blood glucose concentration were not considered; and secondly, the results did not come from different genotypes, sow ages, or suckling intensities. These aspects should be investigated, using HbA1c, the HGI, and TG index as diagnostic tools for glucose intolerance in sows during late pregnancy and lactation. Notably, the strengths of determining glucose intolerance using these methods include the rapid diagnosis and low cost of measuring blood glucose, triglycerides, and HbA1c concentrations. Currently, there are electronic devices on the market that can measure these indicators under field conditions in less than 5 min at an approximate cost of $ 13.00 USD.

## Conclusion

HbA1c, HGI, and TG index tests are viable alternatives for diagnose glucose intolerance in lactating sows and, with this diagnosis, anticipate feeding strategies that maximize feed intake in sows that they will present glucose intolerance. It should be noted that further research is required on the use of these tools (HbA1c, the HGI, and TG index) to diagnose glucose intolerance in sows. Future studies should consider more variables (including feeding frequency, litter size, suckling intensity, sow age, and genotype), a larger sample size, and the evaluation of these factors under commercial production practices.

## Supporting information

S1 File (ZIP)
